# Artificial intelligence in differentiating tropical infections: A step ahead

**DOI:** 10.1371/journal.pntd.0010455

**Published:** 2022-06-30

**Authors:** Shreelaxmi Shenoy, Asha K. Rajan, Muhammed Rashid, Viji Pulikkel Chandran, Pooja Gopal Poojari, Vijayanarayana Kunhikatta, Dinesh Acharya, Sreedharan Nair, Muralidhar Varma, Girish Thunga

**Affiliations:** 1 Department of Pharmacy Practice, Manipal College of Pharmaceutical Sciences, Manipal Academy of Higher Education, Manipal, India; 2 Department of Computer Science & Engineering, Manipal Institute of Technology, Manipal Academy of Higher Education, Manipal, India; 3 Department of Infectious Diseases, Kasturba Medical College, Manipal Academy of Higher Education, Manipal, India; The Rockefeller Foundation, UNITED STATES

## Abstract

**Background and objective:**

Differentiating tropical infections are difficult due to its homogenous nature of clinical and laboratorial presentations among them. Sophisticated differential tests and prediction tools are better ways to tackle this issue. Here, we aimed to develop a clinician assisted decision making tool to differentiate the common tropical infections.

**Methodology:**

A cross sectional study through 9 item self-administered questionnaire were performed to understand the need of developing a decision making tool and its parameters. The most significant differential parameters among the identified infections were measured through a retrospective study and decision tree was developed. Based on the parameters identified, a multinomial logistic regression model and a machine learning model were developed which could better differentiate the infection.

**Results:**

A total of 40 physicians involved in the management of tropical infections were included for need analysis. Dengue, malaria, leptospirosis and scrub typhus were the common tropical infections in our settings. Sodium, total bilirubin, albumin, lymphocytes and platelets were the laboratory parameters; and abdominal pain, arthralgia, myalgia and urine output were the clinical presentation identified as better predictors. In multinomial logistic regression analysis with dengue as a reference revealed a predictability of 60.7%, 62.5% and 66% for dengue, malaria and leptospirosis, respectively, whereas, scrub typhus showed only 38% of predictability. The multi classification machine learning model observed to have an overall predictability of 55–60%, whereas a binary classification machine learning algorithms showed an average of 79–84% for one vs other and 69–88% for one vs one disease category.

**Conclusion:**

This is a first of its kind study where both statistical and machine learning approaches were explored simultaneously for differentiating tropical infections. Machine learning techniques in healthcare sectors will aid in early detection and better patient care.

## Author summary

Distinguishing tropical infections is difficult due to its homogeneous nature from clinical and laboratory presentations among them. This is a first of its kind study where both statistical and machine learning approaches were explored simultaneously for differentiating tropical infections. Dengue, malaria, leptospirosis and scrub typhus were the common tropical infections in our settings as per the need analysis. Better predictors in terms of laboratory parameters and clinical presentations were identified from retrospective analysis and used for the regression and machine learning models. The parameters such as accuracy, true positive rate/sensitivity/recall, false positive rate, precision/positive predictive value, F-measure and ROC area for both the training and validation sets (10-fold cross validation) for all modelling approaches and diseases (One vs One and One vs others) were calculated. All the models observed to have an acceptable range of model performance in differentiating tropical infections. Albumin can be considered as the main parameter in differentiating these tropical infections. These models should be implemented in daily clinical routine practice via mobile or desktop assisted applications or tools.

## 1. Introduction

Tropical infectious diseases influence the health of people in tropical climatic conditions thriving in hot and humid areas. It is more common in developing countries presenting as dengue, malaria, leptospirosis, leishmaniasis, scrub typhus, and rickettsial fever [1,2]. They are the major cause of acute febrile illnesses, a major concern of health to people in endemic regions [3]. Globally, tropical infectious diseases lead to melancholy of more than one billion people’s lives with one-half of deaths annually. Children are more prone to tropical infections and every year, one-third of the infected children die due to malaria [4,5].

Biological factors like residential status (rural vs. urban), living conditions, climatic conditions, nutritional status, environmental factors, poverty, and population density are some of the contributory risk factors [6,7]. Tropical infections are a serious threat to public health, especially in industrialized countries. Dengue is one among the 17 diseases of WHO’s roadmap categorized under neglected tropical diseases which could otherwise be effectively controlled. Almost 2.5 billion people are at risk of contracting the infection and complications could be prevented only by early diagnosis and appropriate treatment on time [8,9]. Saumyen De *et al*. elucidated that most tropical illnesses such as leptospirosis, malaria, scrub typhus, and endemic fever are often confused with dengue fever, especially in a country like India [10]. Malaria was once a major threat globally with tough challenges especially in underdeveloped countries, though the rate of new cases has declined globally by 37% as of 2000–2015. As reported by the researchers of Yale school of public health, about one million people in underdeveloped regions contract leptospirosis with nearly 59,000 deaths especially in Latin America, Asia, Africa, and island nations [11].

The common reason for the uncalled emergence of tropical infections could be due to similar laboratory values, overlapping symptomatology, early asymptomatic presentation, misunderstanding, and delayed diagnosis, which adds up to complications [12]. Predicting its outbreak is a challenging issue due to non-specific clinical presentations of these tropical infectious diseases [13]. The gold standard of diagnosis at an early stage involves specific serological investigations. In the case of common seasonal patterns and overlapping symptoms, a delay in differential diagnosis makes the situation complicated and worsened. Usually, the specific differential test for malaria and dengue takes 24 hours and >24–48 hours for rickettsial fever-like leptospirosis and scrub typhus [14,15].

Detailed history taking, clinical examination, and evaluation of laboratory parameters from the beginning lead to accurate end diagnosis with serological values, though this process is tedious and burdensome to healthcare professionals [16]. With progression in this technological world, machine learning which is a sub-discipline of artificial intelligence (AI) has been playing a significant role in the advancement of healthcare technology [17]. AI has revolutionized the dynamic healthcare industry through its application in various domains like drug discovery, drug repurposing, insilico clinical trials, epidemic outbreak prediction, precision health, diagnosis, prognosis and prediction [18]. It is a fact that, not only lack of appropriate management strategies contributes to high mortality, but also challenges in early detection, risk stratification and severity prediction contributes to it [19].

Thus, there is a need for developing a tool that makes optimal use of distinct symptomatology amongst these infections and distinguishable laboratory parameters for early identification of cause of acute febrile illnesses. Early diagnosis could in addition lead to appropriate antibiotic therapy thus reducing antibiotic resistance along with a better patient care and lesser morbidity or mortality rates in clinical setting [20]. Thus our study aimed to develop a decision making tool to distinguish among tropical infections such as dengue, malaria, leptospirosis and scrub typhus in a tertiary care hospital which is user friendly for early prediction in order to minimize complications due to delayed diagnosis.

## 2. Methodology

### 2.1 Ethical approval

An observational study was conducted at a South Indian tertiary care centre for a period of 9 months from July 2017- March 2018 after obtaining the Institutional Ethics Committee approval (IEC No: 509/2017). A written informed consent was obtained from the participants who are involved in the need analysis (phase I). The data for prediction tool development (phase II) were collected from medical record department retrospectively, hence informed consent was not applicable.

### 2.2 Study design and setting

**Fig 1:**
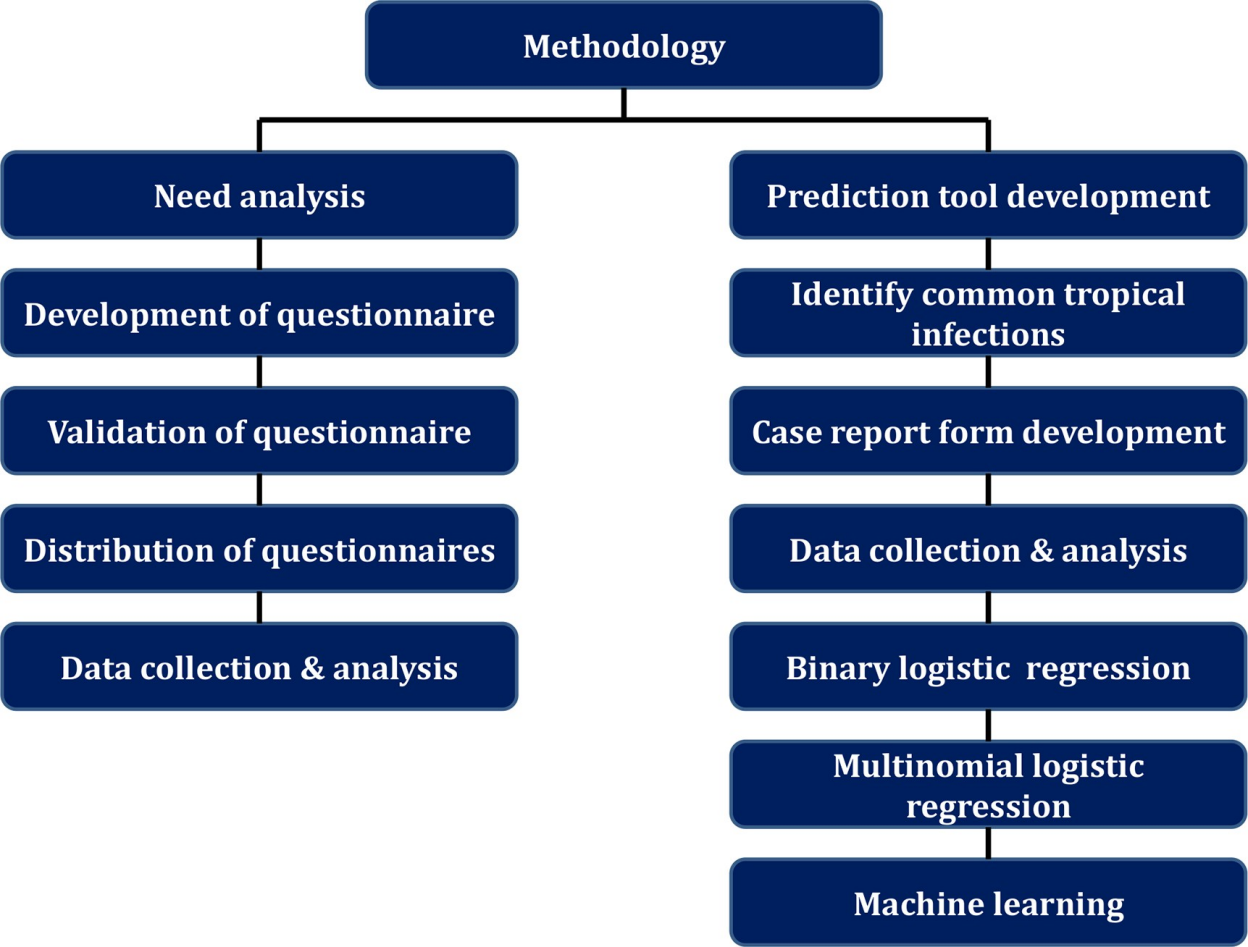
Study flow for need analysis and predication tool development.

This study includes two phases; I) a need analysis phase and a prediction tool development phase. A nine item self-administered questionnaire was developed, validated, distributed and analysed to estimate the need of physicians on differentiating tropical diseases in their setting and components of the same. II) Based on this results a prediction tool was developed with decision tree involving multi-nominal regression analysis and machine learning algorithm. A study flow diagram is given in Fig 1.

### 2.3 Need analysis

#### 2.3.1 Questionnaire development

A literature review assisted nine item self-administered questionnaire was developed to estimate the need of a tool for physicians to differentiate tropical infectious diseases in the hospital. The questionnaire was divided into two parts. The first part was disease specific consisting of 6 questions i.e., frequency of tropical infectious cases, number of cases treated in a week, common observed tropical infections, obstacles in treating tropical infections, challenges in management of infections and the need of tool development. The second part with respect to tool development consisted of 3 questions regarding parameters to be included, format of the tool and additional suggestions.

#### 2.3.2 Questionnaire validation

The content validity of the questionnaire was performed to check its appropriateness and relevance to the study. Six experts including 2 physicians and 4 pharmacy academicians from the study setting validated the questionnaire. Content validity index (CVI) consisted of validation of individual questions and overall scale validity of questionnaire was calculated once all experts completed the validation process; and final validated questionnaire was prepared.

#### 2.3.3 Dissemination of questionnaire and analysis

Validated questionnaire was distributed to physicians or medical students (residents and post graduates) who were involved in treating tropical infections and acute febrile illnesses to assess the need of developing and implementing a decision making tool. Physicians or students who were not currently affiliated in the department of medicine or not treating tropical infections at the hospital were excluded from the study. The need for tool development was explained to them in a one-to-one basis. Relevant data from physician’s feedback on questionnaire was collected which include specific tropical infectious diseases and parameters to be included in decision making tool. The descriptive data was analysed and presented as frequency and percentage.

### 2.4 Prediction tool development

#### 2.4.1 Data collection and statistical analysis

A validated case report form (CRF) was designed based on physician’s feedback in which patient’s data was collected retrospectively. Data was analysed through Statistical package for social sciences (SPSS Inc., version 20.0). The descriptive data were presented as frequency and percentage and continuous variables were represented by mean (SD) or median (range). Only those parameters which were statistically significant (two-sided P≤0.05) with high odds ratio (OR) through multi-nominal logistic regression from clinical and laboratorial parameters were considered for tool development.

### 2.5 Development of the data sets and decision making tool

We aimed to generate a simple scoring system which could differentiate among common tropical infections (dengue, leptospirosis, malaria and scrub typhus) through basic clinical and laboratory results available within few hours of admission in hospitals by which a simple decision tree model could be generated. A total of 800 patients with 200 in each group (dengue, malaria, leptospirosis and scrub typhus) were collected and analysed accordingly. Thirteen variables were selected in order to differentiate among tropical infections. Multinomial logistic regression analysis was performed by dividing the data into two categories i.e., one containing 170x4 cases (training set) and other containing 30x4 cases (validation set with dengue fever as reference category).

### 2.6 Machine learning algorithm

Waikato Environment for Knowledge Analysis (WEKA) software was used for machine learning modelling. Binary classification analysis as well as multi-classification analysis was used for machine learning. Under binary classification two strategies were adopted. Firstly, one vs rest strategy (other) where a single classifier per class is trained with sample of that class as positive and all other samples as negative; Secondly, by taking two diseases at a time i.e., one vs one strategy. Multi-classification involved several algorithms which were based on neural networks, decision tree, random forest, and multinomial regression. The above said models were applied to the same dataset to obtain results accordingly.

## 3. Result

A 9 item validated self- administered questionnaire was designed to determine the demand of physicians on decision making tool in order to differentiate among tropical infections.

### 3.1. Need analysis

#### 3.1.1 Validation of the questionnaire

All 9 items in our questionnaire was found to be relevant and appropriate to our study with a CVI of greater than 0.78. The overall scale CVI (S-CVI) of our questionnaire was appeared to be 0.94.

#### 3.1.2 Dissemination of questionnaire

The validated questionnaire was circulated among 40 physicians and post graduate students in department of medicine which consisted of head of the department (3), associate professor (3), assistant professor (4) senior residents (1) junior residents (14), post graduates (6) and interns (9).

#### 3.1.3. Perspectives of physicians

All the 40 physicians claimed that they often treat tropical infections with an average of 24 cases weekly. Among the diseases observed, dengue (n = 16) was common, followed by scrub typhus (n = 15), malaria (n = 14), leptospirosis (n = 12) and influenza (n = 4). Majority of them (n = 24) faced challenges in treating tropical infections, most of them (n = 34) felt management of symptomatology to be difficult, followed by diagnosis (n = 30). The majority of the participants (n = 35) felt the need for development of decision making tool. Most of them agreed on including laboratorial parameters (n = 34) and clinical presentations (n = 35) as main criterion in the tool and preferred to be in an online app format (n = 36). Based on the perspectives of treating physician, a validated CRF was used to collect the patient data.

### 3.2 Prediction tool development

#### 3.2.1 Demographic characteristics of patients

A total of 800 patients were included with a male domination (68.5%), out of which 9.5% were geriatric population. The mean age of the study population was 39.1±14.9 years. Geographical spreading showed higher rates of leptospirosis and scrub typhus from north side of the state, whereas dengue and malaria rates were higher in south side. The demographic details and clinical parameters of each disease is given in **Table 1**.

**Table 1:** Demographic details and clinical parameters of each disease categories.

Parameter	Dengue	Malaria	Scrub typhus	Leptospirosis
Age (Mean±SD), years	34.1±13.1	35.5±13.9	45.1±14.9	41.8±14.7
Gender (male/female)	139/61	162/38	113/87	134/66
Abdominal pain (n)	35	19	42	27
Sweating (n)	3	17.5	1	3
Arthralgia (n)	15.5	9	5.5	2.5
Myalgia (n)	24.5	16	21	13
Mobilliform rash (n)	0	0	21.5	2.5
Conjunctival congestion (n)	3.5	2	4	31
Maculopapular rash(n)	22	2.5	0.5	0.5
Yellowish urine (n)	2	19	17.5	8
Yellowish skin (n)	0	17.1	0	1.5
Hepatomegaly (n)	13.5	17.5	0	0.5
Weight loss (n)	0	1	0.5	21.5
Decreased urine output (n)	1.5	17.5	2	4
Splenomegaly (n)	0	26	0	0
Lymphadenopathy (n)	1.5	0.5	25.5	0
Pallor (n)	1	32	5.5	6

Based on the clinical presentation of all four tropical infections, a multinomial regression analysis was performed to identify the significant factors in predicting score of each disease.

### 3.3 Multinomial regression analysis and predictability score of each disease

The multinomial logistic regression analysis based on nine scoring models predicted scores of dengue, malaria, leptospirosis and scrub typhus to be 60.7%, 62%, 66% and 38% respectively. Nine variables with highest OR and p<0.05 were chosen. They were categorized on the basis of arbitrary cut offs as myalgia (present or absent), arthralgia (present or absent), abdominal pain (present or absent), urine output (normal or decreased), total bilirubin (0–1.6; 1.6–3.2; 3.2mg/dl and above), sodium level (100-140ml; 140ml and above), albumin level (0–3.4; 3.5mg/dl and above), red blood cell (1500000–3500000; 3600000–4500000; 4600000–5500000; 5600000cells/cumm and above), lymphocytes (10–20; 21–40; 40 cells/cumm and above), haematocrit (25–35; 35–45; 45–55%), platelets (5000–50,000; 50,000–1,00,000; 1,00,000–1,50,000; 1,50,000–4,50,000; 4,50,000cells/cumm and above) and erythrocyte sedimentation rate (0–22; 22–30; 30mm/hr and above). The predictability score of each disease category is provided in Table 2.

**Table 2:** Predictability score of each disease category.

Disease	Variable	Odds Ratio (OR)	95% Confidence Interval (95% CI)
**MALARIA**	**Sodium Level (ml)**		
	100–140 ml	3.108	1.020–94
	140ml and above		
	**Total Bilirubin Level (mg/dL)**		
	0–1.6	.065	.014-.302
	1.6–3.2	.679	.118–3.891
	3.2 and above		
	**Albumin (mg/dL)**		
	0–3.4	.683	.313–1.490
	3.5 and above		
	**Lymphocytes (cells/cumm)**		
	10–20	3.432	1.447–8.136
	21–40	2.062	.862–4.933
	40 and above		
	**Platelets (cells/cumm)**		
	5000–50000	.385	.162-.96
	50000–100000	1.086	.492–2.397
	100000–150000	.832	.347–1.997
	150000–450000		
	**Abdominal pain**		
	Present	.551	.2441.274
	Absent	.	.
	**Athralgia**		
	Present	.595	.252–1.404
	Absent		
	**Myalgia**		
	Present	1.001	.482–2.081
	Absent	.	.
	**Urine output**		
	Decreased	.250	.091–0.607
	Normal		
**SCRUB TYPHUS**	**Sodium Level (ml)**		
	100–140 ml	1.451	.457–4.610
	140ml and above	.	.
	**Total Bilirubin Level (mg/dL)**		
	0–1.6	.051	.011-.237
	1.6–3.2	.392	.068–2.266
	3.2 and above		
	**Albumin (mg/dL)**	.	.
	0–3.4	12.628	6.204–25.703
	3.5 and above		
	**Lymphocytes (cells/cumm)**		
	10–20	1.718	.709–4.161
	21–40	1.546	.629–3.807
	40 and above		
	**Platelets (cells/cumm)**		
	5000–50000	.030	.012–0.076
	50000–100000	.108	.048–0.246
	100000–150000	.202	.086–0.477
	150000–450000		
**LEPTOSPIROSIS**	**Abdominal pain**		
	Present	1.263	.570–2.799
	Absent		
	**Athralgia**		
	Present	.127	.036-.452
	Absent	.	.
	**Myalgia**		
	Present	1.453	.659–3.206
	Absent		
	**Urine output**		
	Decreased	2.706	.612–11.504
	Normal		
	**Sodium Level (ml)**		
		
	100–140 ml	.646	.211–1.916
	140ml and above	.	.
	**Total Bilirubin Level (mg/dL)**		
	0–1.6	.027	.006-.124
	1.6–3.2	.210	.036–1.208
	3.2 and above		
	**Albumin (mg/dL)**		
	0–3.4	11.624	5.523–24.261
	3.5 and above		
	**Lymphocytes (cells/cumm)**		
	10–20	41.490	8.065–213.448
	21–40	12.801	2.412–67.947
	40 and above		
	**Platelets (cells/cumm)**		
	5000–50000	.235	.097-.567
	50000–100000	.147	.059-.366
	100000–150000	.208	.078–553
	150000–450000		
**DENGUE**	**Abdominal pain**		
	Present	.925	.386–2.218
	Absent	.	.
	**Athralgia**		
	Present	.036	.006-.227
	Absent		
	**Myalgia**		
	Present	1.146	.479–2.738
	Absent	.	.
	**Urine output**		
	Decreased	1.030	.275–3.437
	Normal		

### 3.4 Multinomial logistic regression analysis for significant parameters among scrub typhus, malaria, and leptospirosis with dengue disease category as reference

**Fig 2:**
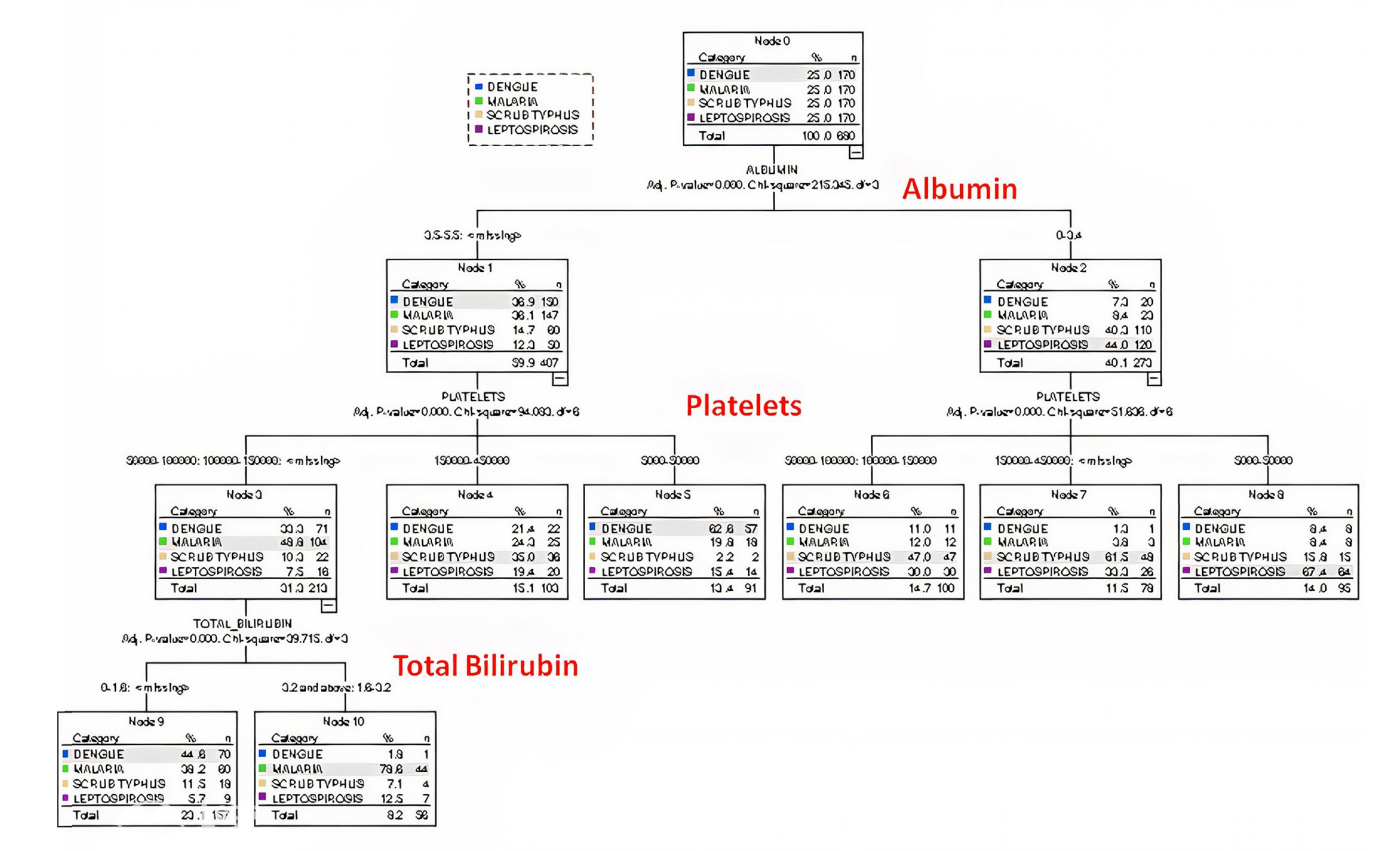
Decision tree model.

On the basis of various characteristics, a decision tree model was developed. According to the tree, albumin could be considered as the main parameter to differentiate among 4 tropical infections followed by platelets and bilirubin levels (p<0.05). The decision tree model is depicted in Fig 2.

### 3.5 Machine learning modelling

WEKA machine learning tool was applied to test binary (one disease at a time) and multi-class (all the four diseases) classification. Multi-class classification based on the random forest (multiple decision trees), neural network (back propagation), decision tree and adaboost (logistic regression base class) showed an average accuracy of 55–60%. Binary classification (one vs. rest) using logistic regression showed an estimated accuracy of 79–84%. Binary allocation with logistic regression by sampling two diseases at a time (one vs. one) showed an accuracy of 69–88%. Attribute visualization showed value overlapping among diseases with no attributes helping in classifying diseases. Correct classified instances by decision tree were 50.37%, random forest (56.62%), multinomial logistic regression (59.75%), adaboost (59.75%) and multilayer perception (55.88%). Binary classification of dengue vs. others showed exact classified instances of 83.75%, malaria vs. others (79.12%), scrub typhus vs. others (79.41%), leptospirosis vs. others (82.72%), dengue vs. malaria (69.11%), leptospirosis vs. scrub typhus (70.59%), dengue vs. scrub typhus (80.88%), dengue vs. leptospirosis (84.56%), malaria vs. leptospirosis (75.73%) and malaria vs. scrub typhus (87.50%), respectively. The summary (accuracy) of binary classification is provided in Table 3. Attribute visualization for each disease and the model classifications through machine learning modelling is presented in S1 File. The parameters such as true positive rate/sensitivity/recall, false positive rate, precision/positive predictive value, F-measure and receiver operating characteristic (ROC) area for both the training and validation sets (10-fold cross validation) for all modelling approaches and diseases (One vs One and One vs others) also calculated. The data visualization and parameters with respect to the disease variable is provided in S2 File.

**Table 3:** Summary (accuracy) of binary classification.

	OTHERS	MALARIA	SCRUB TYPHUS	LEPTOSPIROSIS
DENGUE	83.8%	69.1%	80.9%	84.6%
MALARIA	79.1%	X	75.7%	87.5%
SCRUB TYPHUS	79.4%	X	X	70.6%
LEPTOSPIROSIS	82.7%	X	X	X

### Effect of age on model performance

To study the effect of age on model performance, the data set were divided into four groups, with equi-frequency such as 18–26.5; 26.5–36.5; 36.5–50.5; and 50.5–80. The classifiers such as random forest, multinomial logistic regression and multi-layer perceptron were applied. The accuracy of the model based on age classification was 67 to 71%. We did not observe any changes in the accuracy as well as other parameters such as true positive rate/sensitivity/recall, false positive rate, precision/positive predictive value, F-measure and ROC area of the model based on the age. The results are provided in S3 File.

## 4. Discussion

Tropical infections such as dengue, malaria, scrub typhus, leptospirosis, rickettsial fever, and leishmaniasis are the prevailing reasons for febrile illness now-a-days especially in endemic region [21]. Similar laboratory parameter for evaluation of these infections and extending symptomatology makes it difficult to be diagnosed at starting stage. Dengue is considered as the cause of febrile illness, even in case of scrub typhus and rickettsial fever. Similarly in south-east Asian region, malaria and leptospirosis are the other common cause of febrile illness. Mostly, these diseases present with non-specific clinical characters which makes it difficult to differentiate among each other [22]. Hence, development of a tool which could differentiate within these diseases based on the initial presentation is beneficial for physicians for early diagnosis and effective management.

In India especially many parts of southern India, the most common and recurrent presumptive diagnosis among those hospitalized with undifferentiated fever pattern is dengue, malaria, leptospirosis and scrub typhus. Many attempts have been taken by researchers in predicting among these illnesses [23,24]. Mitra *et al*., elaborated on differentiating clinical and laboratory parameters in patients with scrub typhus and dengue. They included age (>30 and ≤30 years), haemoglobin (≤14 and >14 g/dL), total white blood cells count (<4000, 4001–7000 and >7000 cells/cumm), oxygen saturation (>90%, ≤90%), total bilirubin (≤2 and >2 mg/dL) altered sensorium (present or absent) and serum glutamic-oxaloacetic transaminase (≤200 IU/dL) and >200) which were all significant in predicting differences among these two illnesses [25]. Varma *et al*., compared between dengue and leptospirosis and reported muscle tenderness, leukocytopenia, elevated erythrocyte sedimentation rate, oliguria, acute renal failure, icterus, anaemia, thrombocytopenia and hypoalbuminaemia to be common in leptospirosis compared to dengue [26]. Mortality ratio of leptospirosis to dengue was 18:1. However, no model has been developed yet based on multinomial logistic regression analysis or machine learning modelling which better differentiates among dengue, malaria, scrub typhus and leptospirosis, though there were attempts to find out differentiating features among these diseases.

In our study, we considered dengue, malaria, scrub typhus and leptospirosis as it is the most prevailing tropical infection in our setting with similar clinical presentation which makes them difficult to differentiate at early stage. Along with this, the inference from our pilot survey among medical professional who deal with tropical infection helped us to go ahead with the above infections. We retrospectively collected laboratory parameters of 800 (200 in each group) patients and those factors with highest OR (p<0.05) were considered for the model development. These factors includes four clinical presentations namely i) abdominal pain (present/absent), ii) arthralgia (present/absent), iii) myalgia (present/absent), iv) urine output (decreased/normal); and five laboratory parameters namely v) sodium level (100–140 ml, 140ml and above), vi) total bilirubin level (0–1.6, 1.6–3.2 and ≤3.2mg/dL), vii) albumin (0–3.4, ≤3.5 mg/dL), viii) lymphocytes (10–20, 21–40, ≤40 cells/cumm) and ix) platelets (5000–50000, 50000–100000, 100000–150000 and 150000–450000 cells/cumm).

Multinomial logistic regression and decision tree analysis inferred that albumin could be considered as the main parameter to differentiate among 4 tropical infections followed by platelets and bilirubin levels (p<0.05). These findings were in accordance with the models developed by Mitra *et al*., and Varma *et al*., [25,26]. On the basis of multi-nominal logistic regression, dengue showed 60.7%, leptospirosis showed 66%, malaria showed 62% and scrub typhus showed only 38% predictability, respectively. Recent studies expanded the machine learning applications to the all aspects of medicine and associated fields. Davi C et al., [27] used genome markers to identify individuals at high risk for developing the severe dengue phenotype even in uninfected conditions. Another study by McLaughlin M et al., [28] demonstrated using the malaria rapid diagnostic test "truth" data along with digital mHealth platform clinical assessments and clinical data for a better identification of children with malaria among those with febrile illness.Binary classification machine learning models i.e., one vs. rest showed an average predictability of 79–84% while one disease vs. another showed a score of 69–88%. Multi-classification models such as neural network, decision tree, multinomial regression and random forest plot models showed a predictability score of 55–60%. This could be due to overlapping symptoms and lower accurate arrangement of laboratory and other parameters. In comparison with other studies, our study intended for a dual approach i.e., to establish a model based on machine learning and multinomial logistic regression analysis. In addition we included 4 different tropical diseases along with laboratory and clinical parameters to minimize the chances of misclassification bias. The sample size of our study was also relatively high. In addition to this, the effect of age on model performance was analysed to avoid the confounding effect of age and we did not find any change in model performance based on the age [29,30,31].

Future studies should be conducted to provide more insights towards the application of this study through developing artificial intelligence or computer assisted technology in daily clinical practice. This will help to analyse the credibility of our findings and upgrade further based on the clinical scenario. Also, future studies could focus on a broad aspects of the disease other than parameters considered in our study based on a better knowledge on geographical distribution.

Limitations of our study included the retrospective data collection due to which clinical parameters during initial visits at emergency department or clinic (at outpatient setting) could not be recorded. The findings from this single centred data cannot be generalized to other part of the world as the nature and presentation of tropical diseases varies from location to location. As we used WEKA software for machine learning, it doesn’t provide the parameters such as true negative, false negative and specificity. However, the parameters like accuracy, true positive rate/sensitivity/recall, false positive rate, precision/positive predictive value, F-measure and ROC area was calculated.

## 5. Conclusion

Technology integrated healthcare helps much towards early diagnosis potentiating better quality of life. There is a strong need for physicians in the development of a tool which differentiates tropical infections. Early diagnosis of tropical infections helps in further improving patient care. Our study is the first of its kind where both machine learning and statistical techniques were applied to develop a model in tropical infectious diseases which need to be studied further in implementation level.

## Supporting information

S1 File Model classifications through machine learning modelling. (DOCX)

S2 File The data Visualization and parameters with respect to the disease variable. (DOCX)

S3 File The effect of age on model performance. (DOCX)
