# Supplementary material for: Artificial intelligence in differentiating tropical infections: A step ahead
Source: PLoS Negl Trop Dis. 2022 Jun 30;16(6):e0010455. doi: 10.1371/journal.pntd.0010455 (PMC9246149; doi:10.1371/journal.pntd.0010455)
Supplement: S1 File — (DOCX) [file pntd.0010455.s001.docx]

**Supplementary file S1: Machine learning models through WEKA software**

**A. Multi-class classification**

**Attribute visualization**


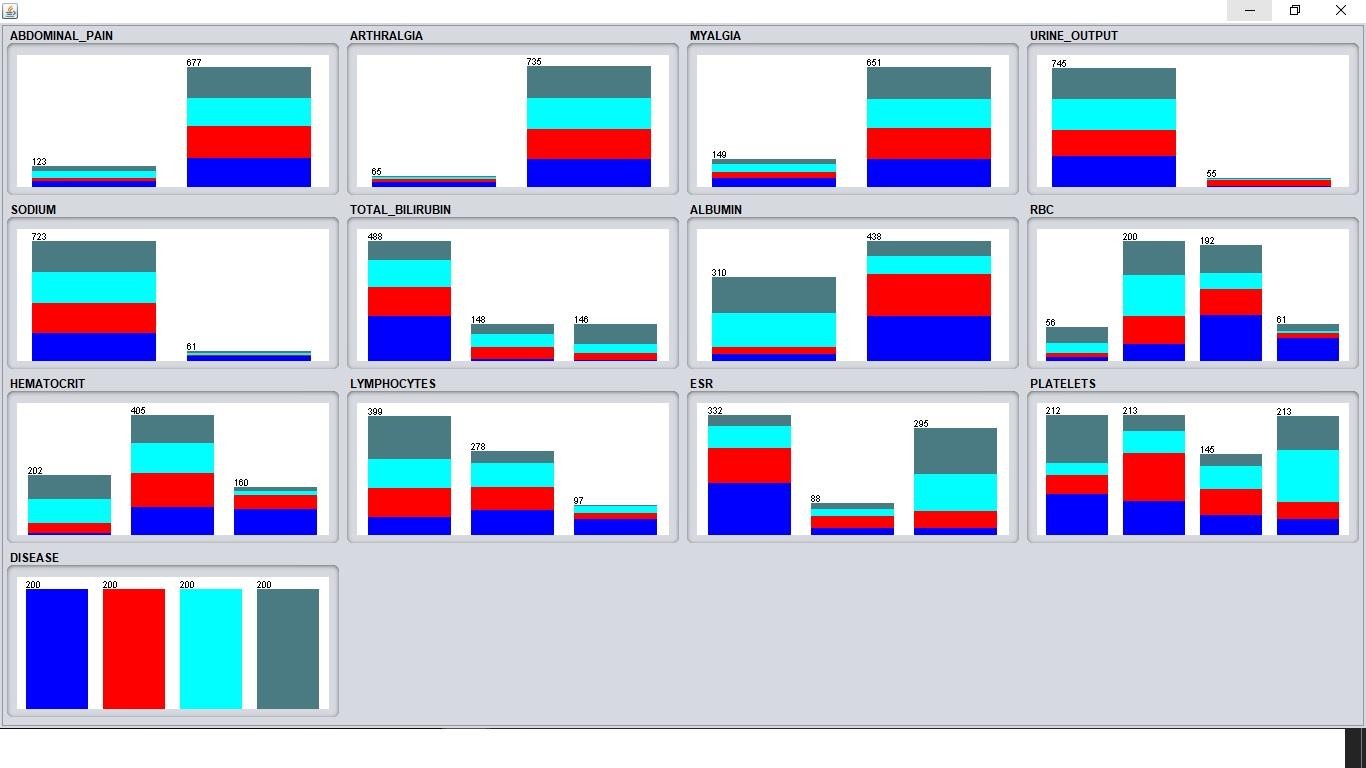
Color Coding:

| **DARK BLUE** = DENGUE |
| --- |
| **RED** =MALARIA |
| **LIGHT BLUE**=SCRUB TYPHUS |
| **GREEN**=LEPTOSPIROSIS |

**1. Decision tree**


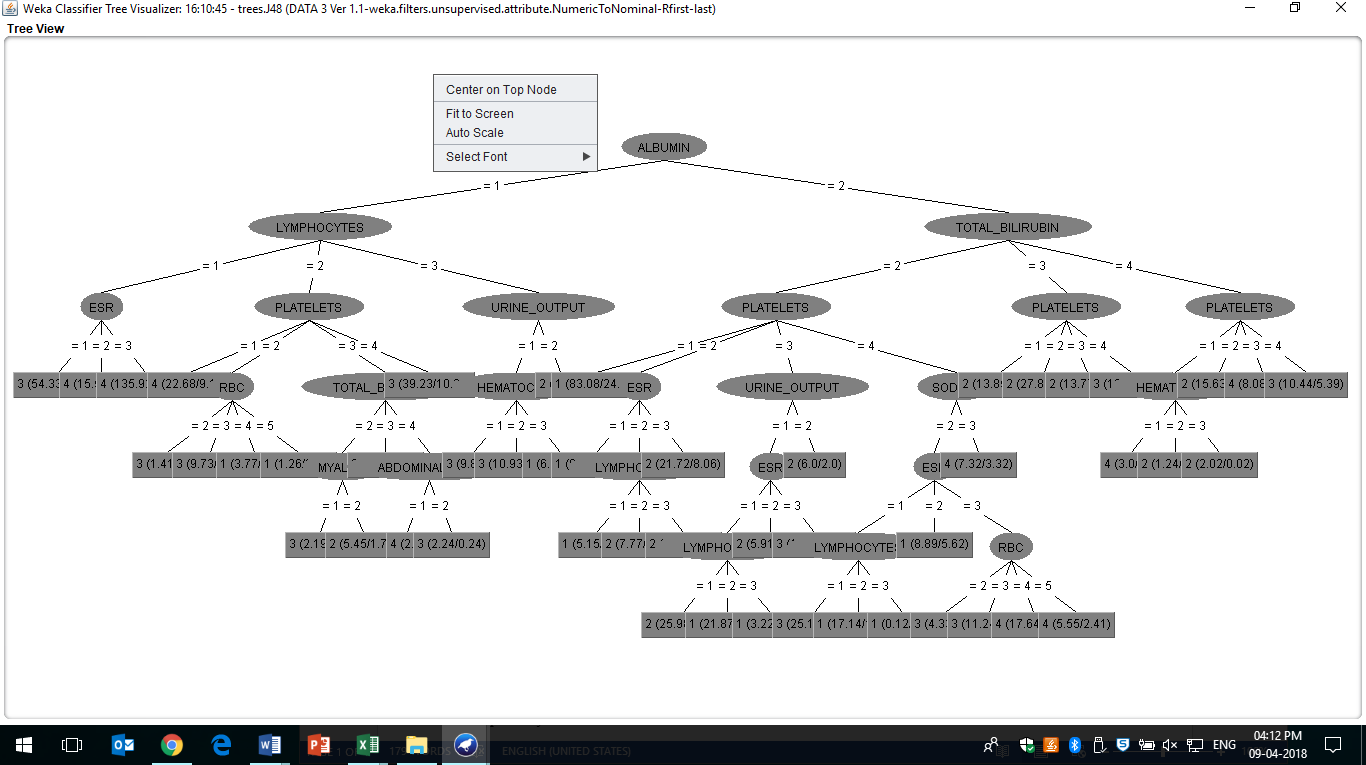


| Correctly Classified Instances | 137 | **50.37 %** |
| --- | --- | --- |
| Incorrectly Classified Instances | 135 | 49.63 % |
| Total Number of Instances | 272 |  |

### 2. Random Forest (uses multiple decision trees based upon subset of data set)

Correctly Classified Instances 154 **56.62 %**

Incorrectly Classified Instances 118 43.38 %

Total Number of Instances 272

### 3. Multinomial Logistic Regression

| Correctly Classified Instances | 478 | **59.75 %** |
| --- | --- | --- |
| Incorrectly Classified Instances | 322 | 40.25 % |
| Kappa statistic | 0.4633 |  |
| Mean absolute error | 0.255 |  |
| Root mean squared error | 0.3631 |  |
| Relative absolute error | 68.0003 % |  |
| Root relative squared error | 83.8621 % |  |
| Total Number of Instances | 800 |  |

**4. Multilayer Perceptron(Back Propogation Neural Network)**

| Correctly Classified Instances | 152 | **55.88 %** |
| --- | --- | --- |
| Incorrectly Classified Instances | 120 | 44.12 % |
| Kappa statistic | 0.4144 |  |
| Mean absolute error | 0.2272 |  |
| Root mean squared error | 0.4315 |  |
| Relative absolute error | 60.5099 % |  |
| Root relative squared error | 99.5035 % |  |
| Total Number of Instances | 272 |  |

**5. Adaboost** ( boosting Multinominal Logistic regression classifier(s))

| Correctly Classified Instances | 478 | **59.75 %** |
| --- | --- | --- |
| Incorrectly Classified Instances | 322 | 40.25 % |
| Kappa statistic | 0.4633 |  |
| Mean absolute error | 0.3464 |  |
| Root mean squared error | 0.4049 |  |
| Relative absolute error | 92.3654 % |  |
| Root relative squared error | 93.4992 % |  |
| Total Number of Instances | 800 |  |

**B. Binary Classification**


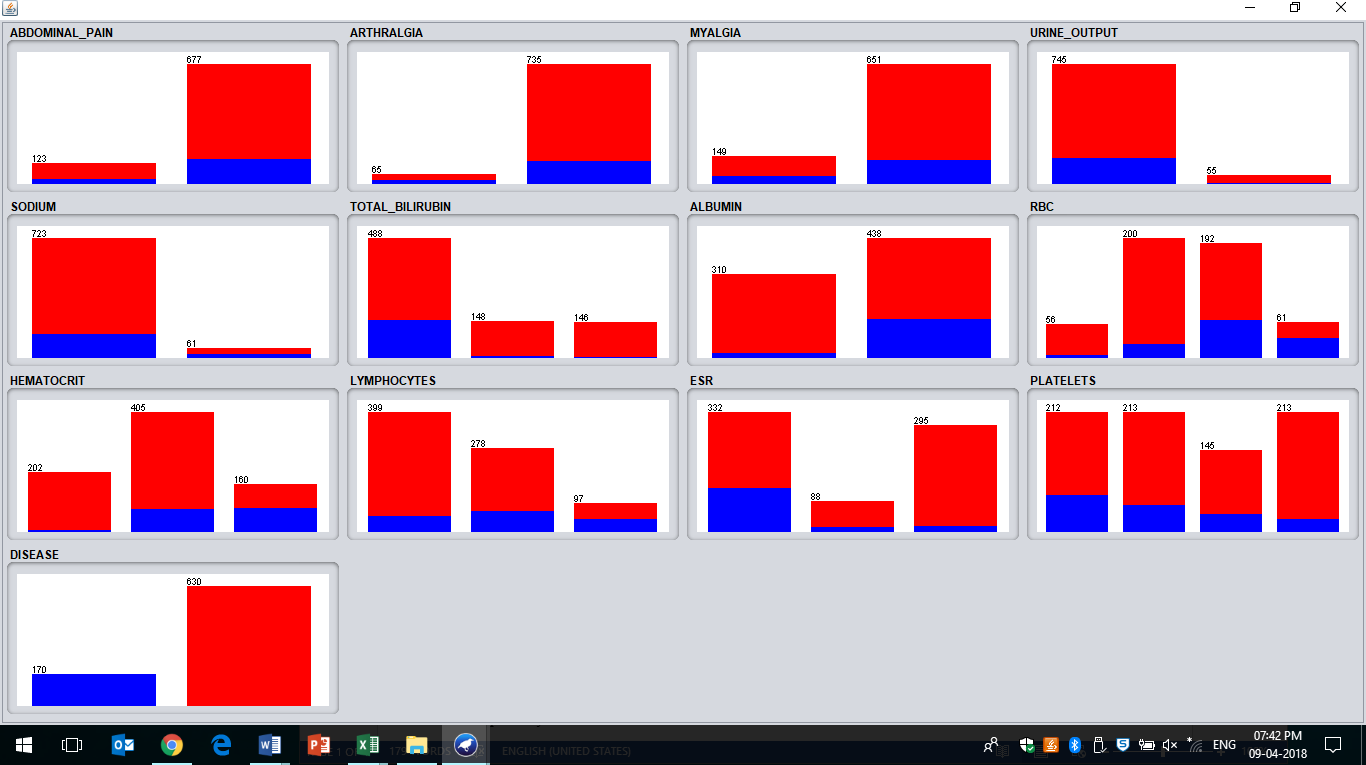
**1. Dengue vs. Others**

| Correctly Classified Instances | 670 | **83.75** | **%** |
| --- | --- | --- | --- |
| Incorrectly Classified Instances | 130 | 16.25 | % |
| Total Number of Instances | 800 |  |  |


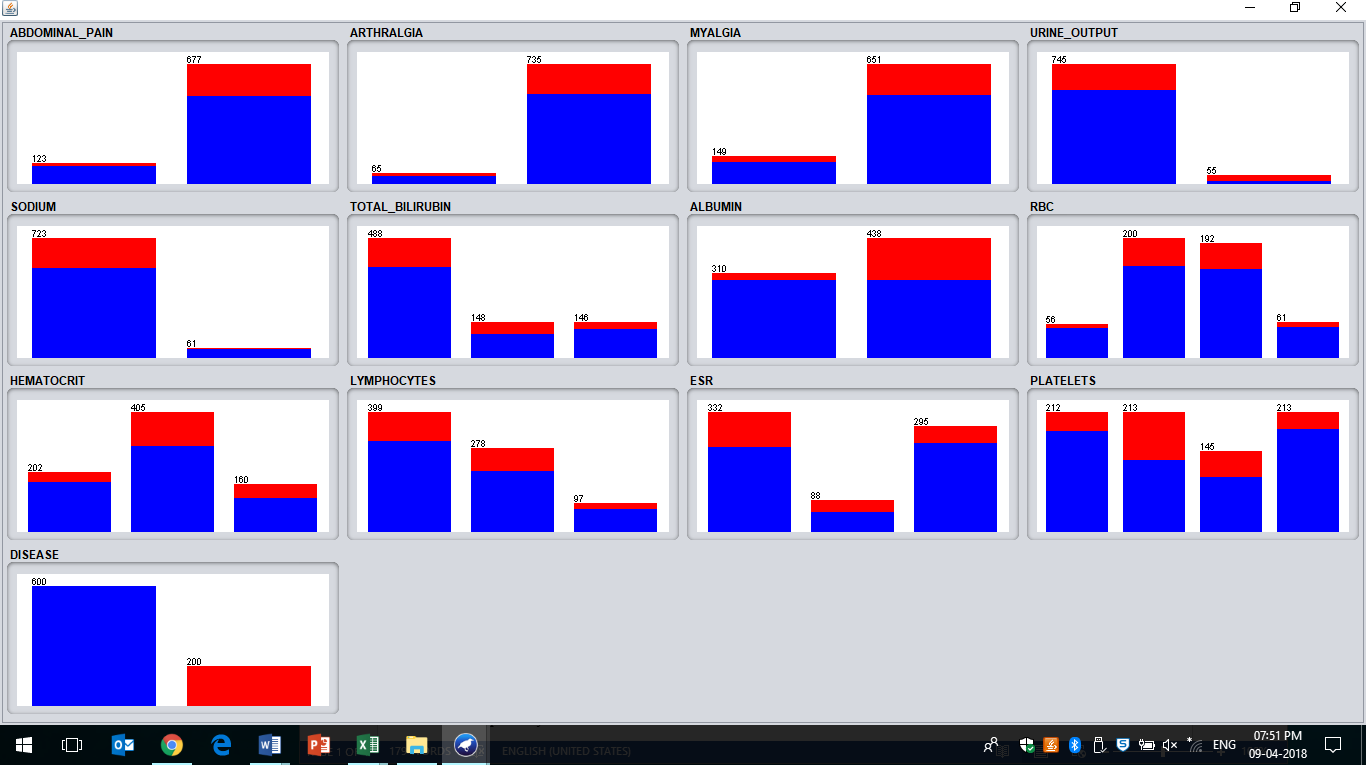
**2. Malaria vs. Others**

Correctly Classified Instances 633 **79.12 %**

Incorrectly Classified Instances 167 20.87 %

Total Number of Instances 800

**3. Scrub Typhus vs. Others**


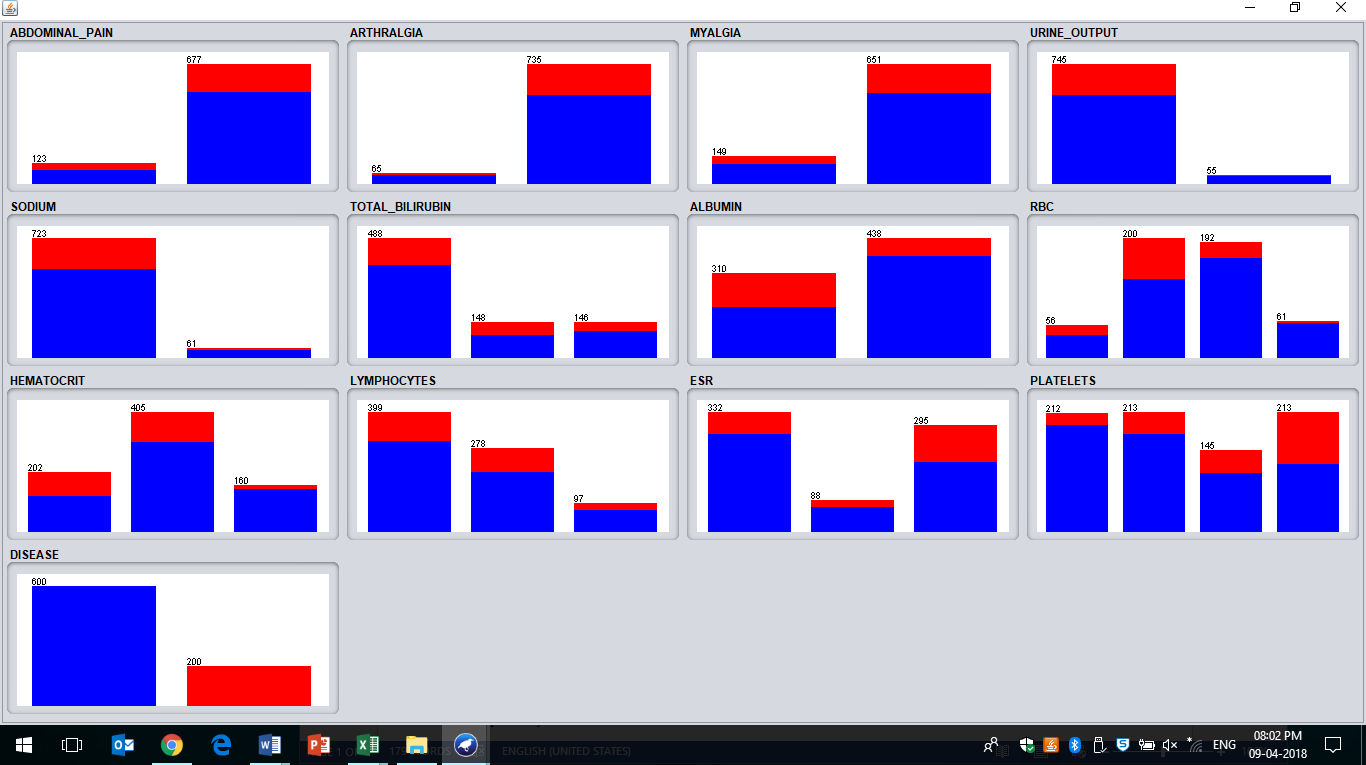


| Correctly Classified Instances | 216 | 79.41 % |
| --- | --- | --- |
| Incorrectly Classified Instances | 56 | 20.59% |
| Total Number of Instances | 272 |  |

**4. Leptospirosis vs. Others**


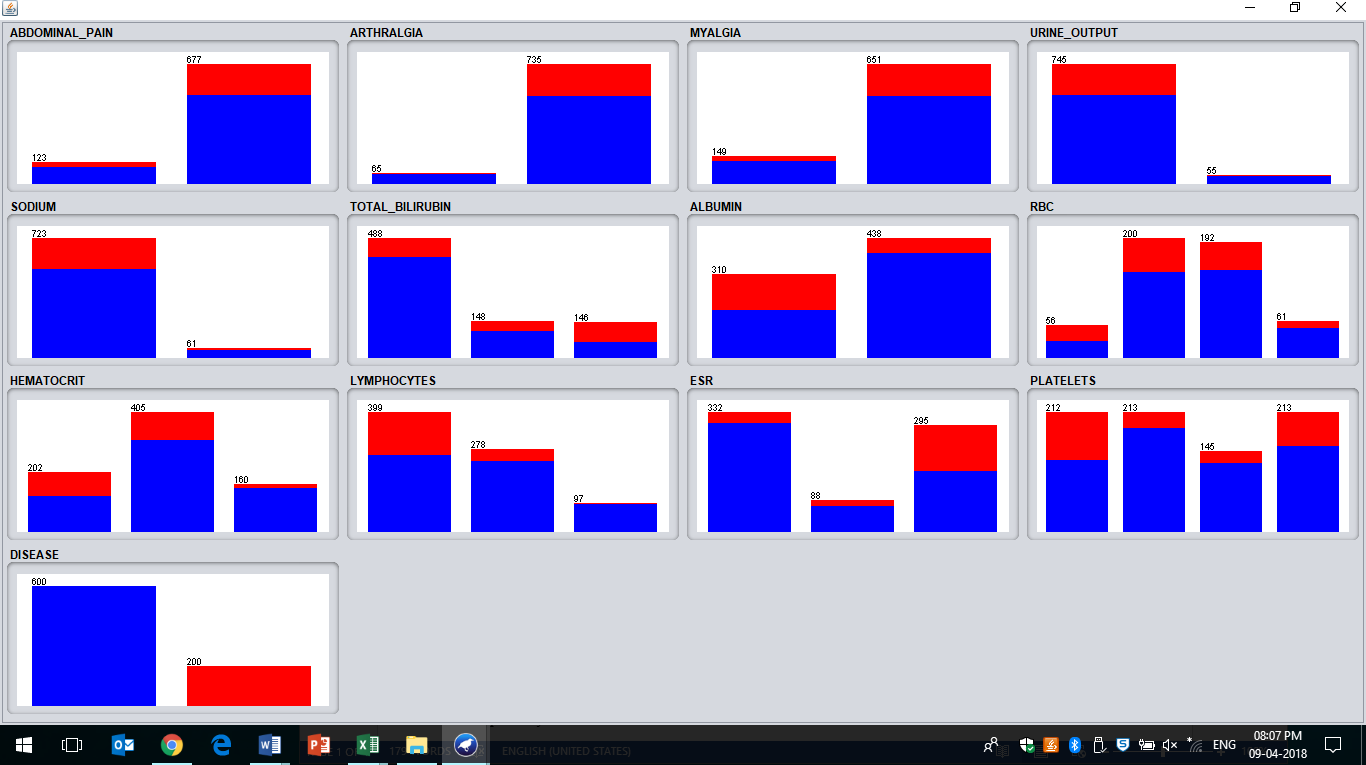


| Correctly Classified Instances | 225 | 82.72 % |
| --- | --- | --- |
| Incorrectly Classified Instances | 47 | 17.28 % |
| Total Number of Instances | 272 |  |

**5. Dengue vs. Malaria**


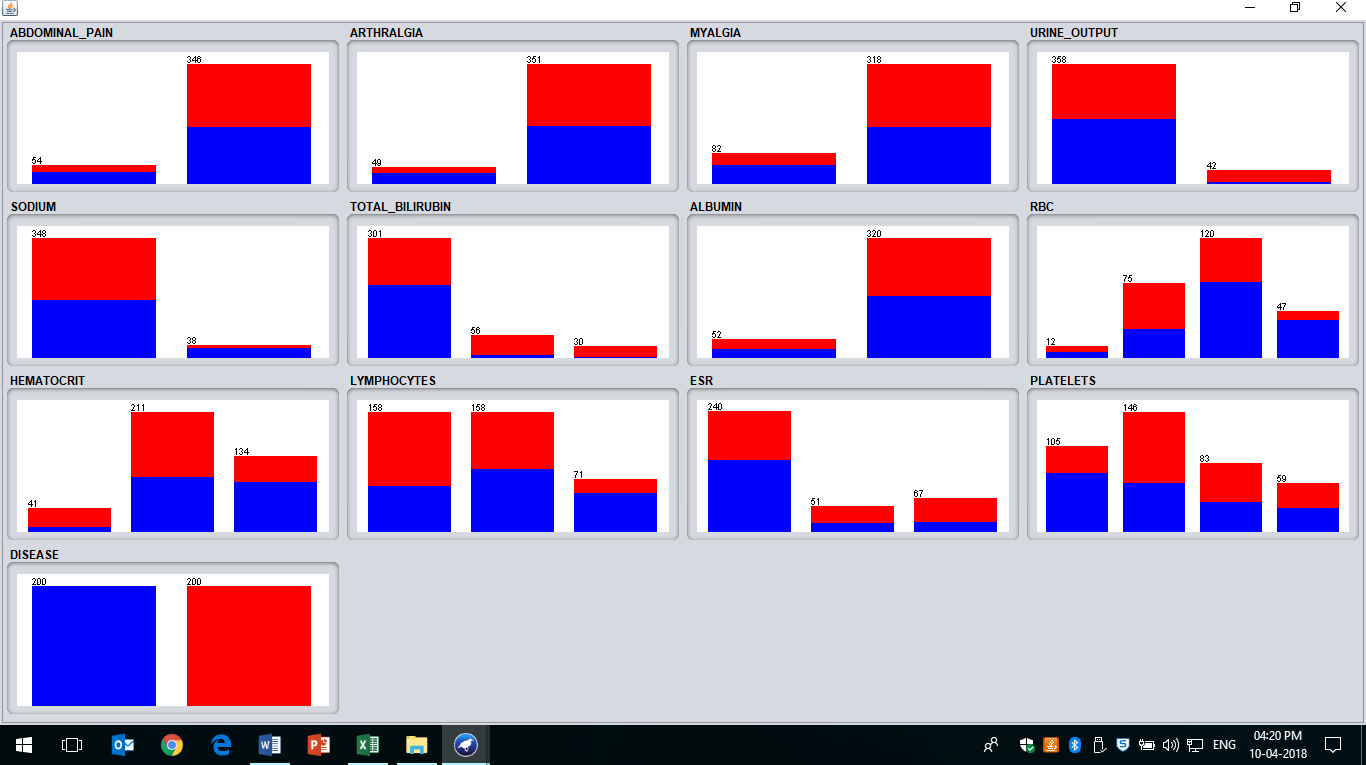


| Correctly Classified Instances | 94 | 69.12 % |
| --- | --- | --- |
| Incorrectly Classified Instances | 42 | 30.88 % |
| Total Number of Instances | 136 |  |


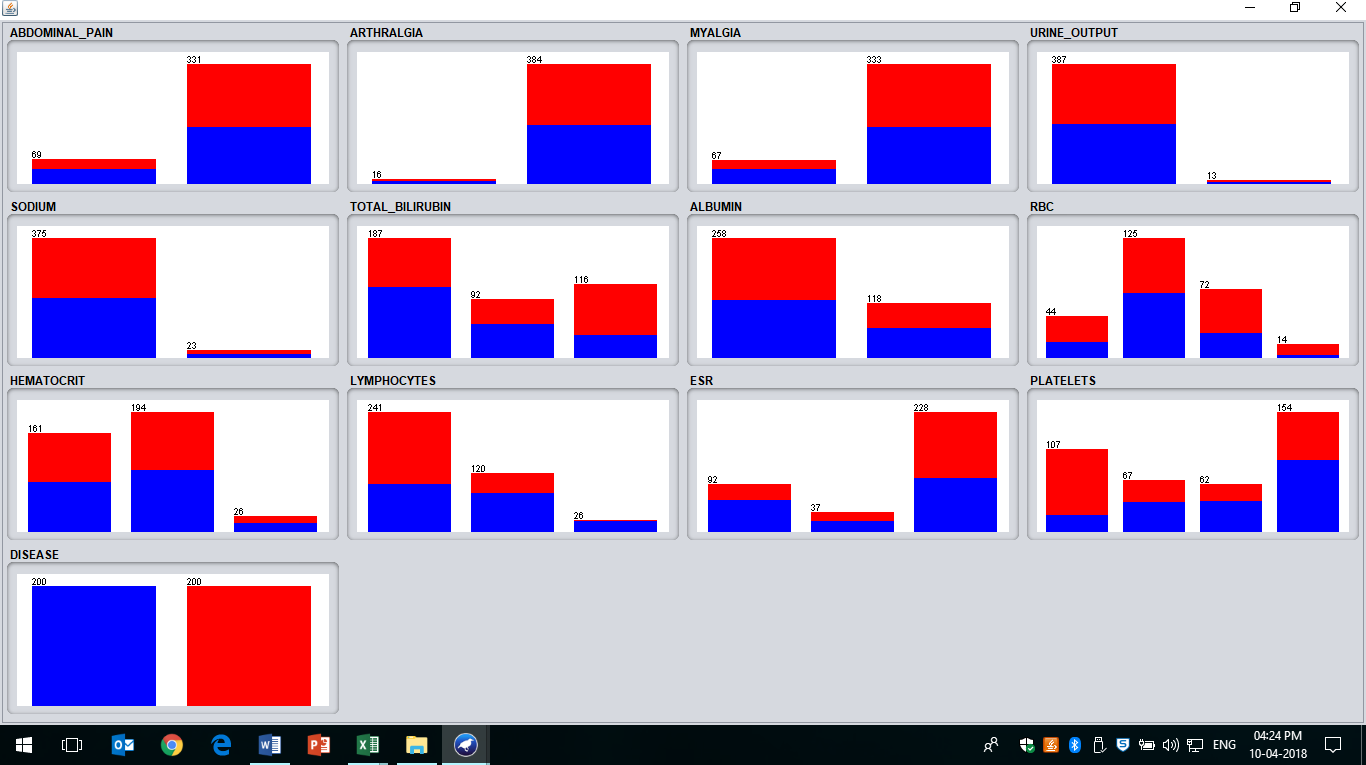
**6. Leptospirosis vs. Scrub typhus**

| Correctly Classified Instances | 96 | **70.59 %** |
| --- | --- | --- |
| Incorrectly Classified Instances | 40 | 29.41 % |

Total Number of Instances 136


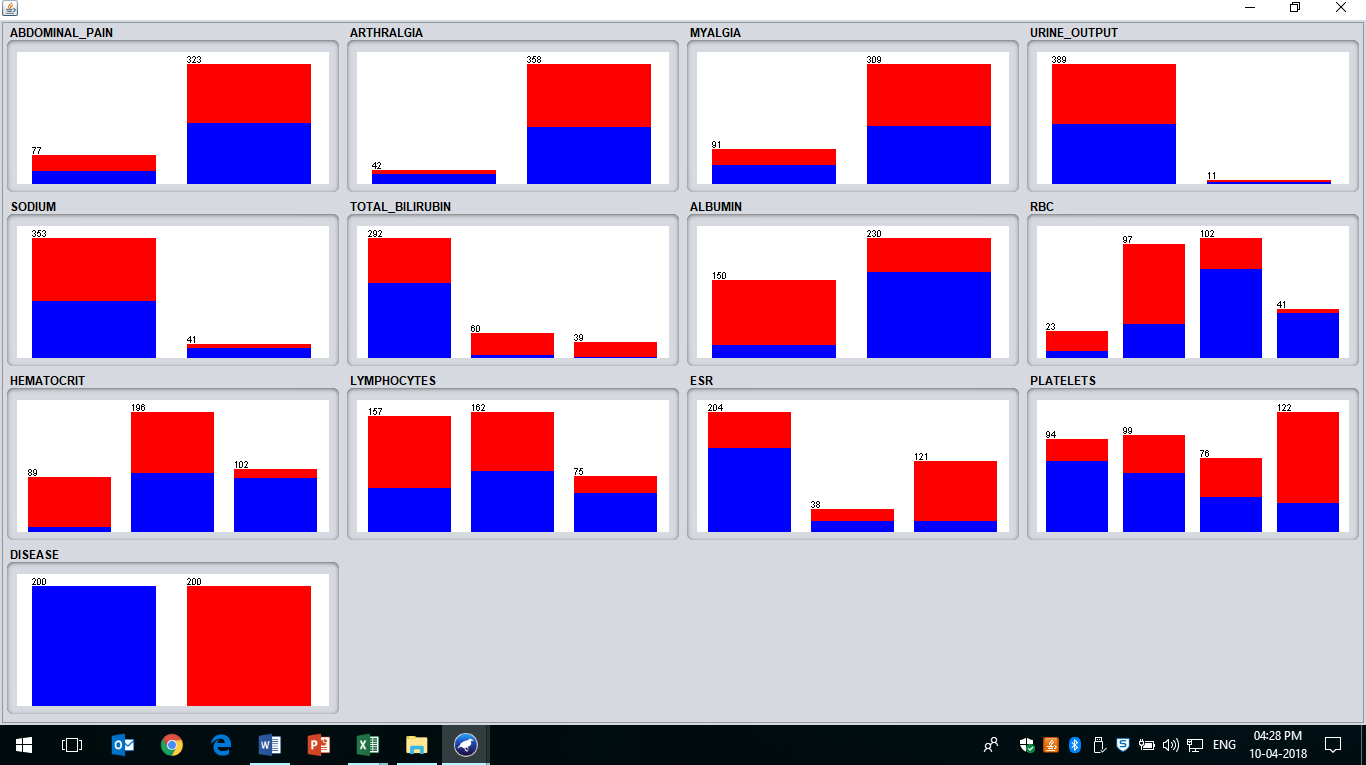
**7. Dengue vs. Scrub Typhus**

| Correctly Classified Instances | 110 | **80.88 %** |
| --- | --- | --- |
| Incorrectly Classified Instances | 26 | 19.12 % |
| Total Number of Instances | 136 |  |

**8. Dengue vs. Leptospirosis**


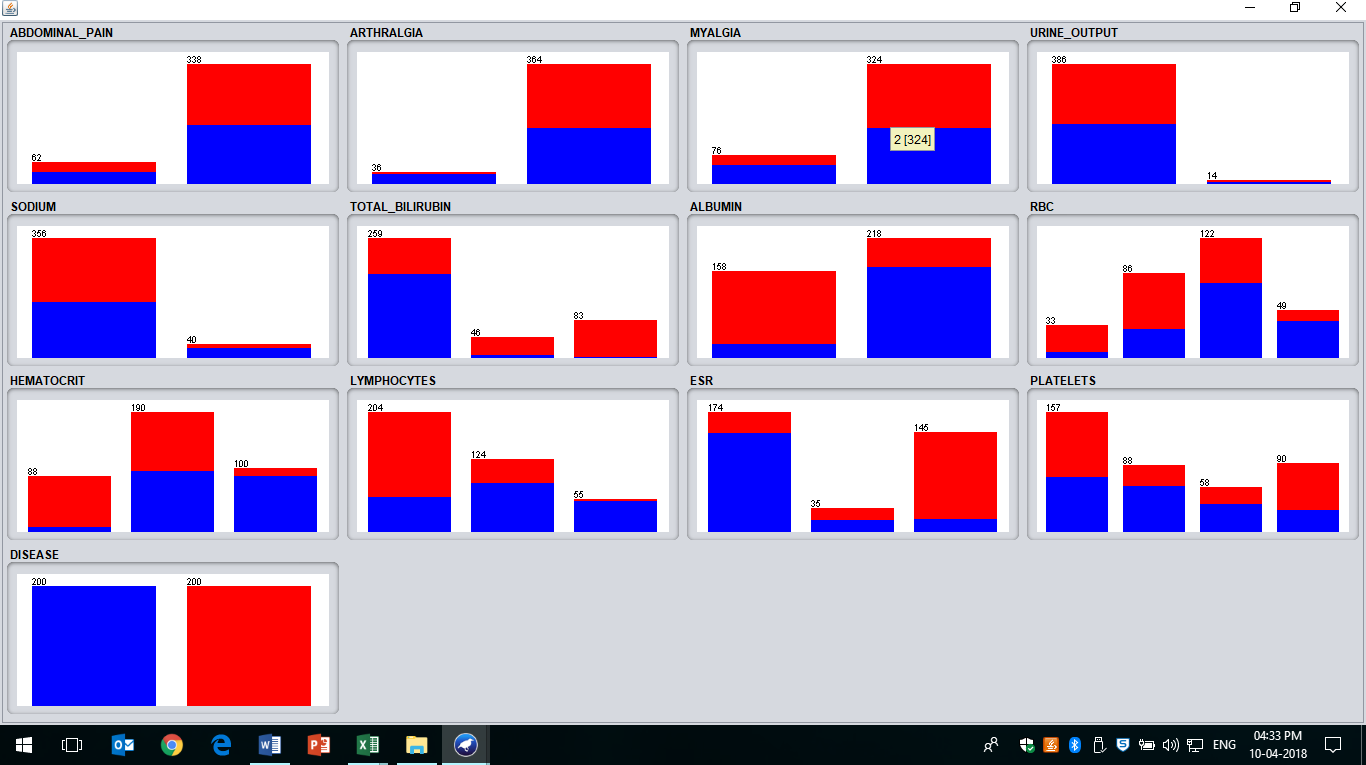


| Correctly Classified Instances | 115 | **84.56 %** |
| --- | --- | --- |
| Incorrectly Classified Instances | 21 | 15.44 % |
| Total Number of Instances | 136 |  |

**9. Malaria vs. Leptospirosis**


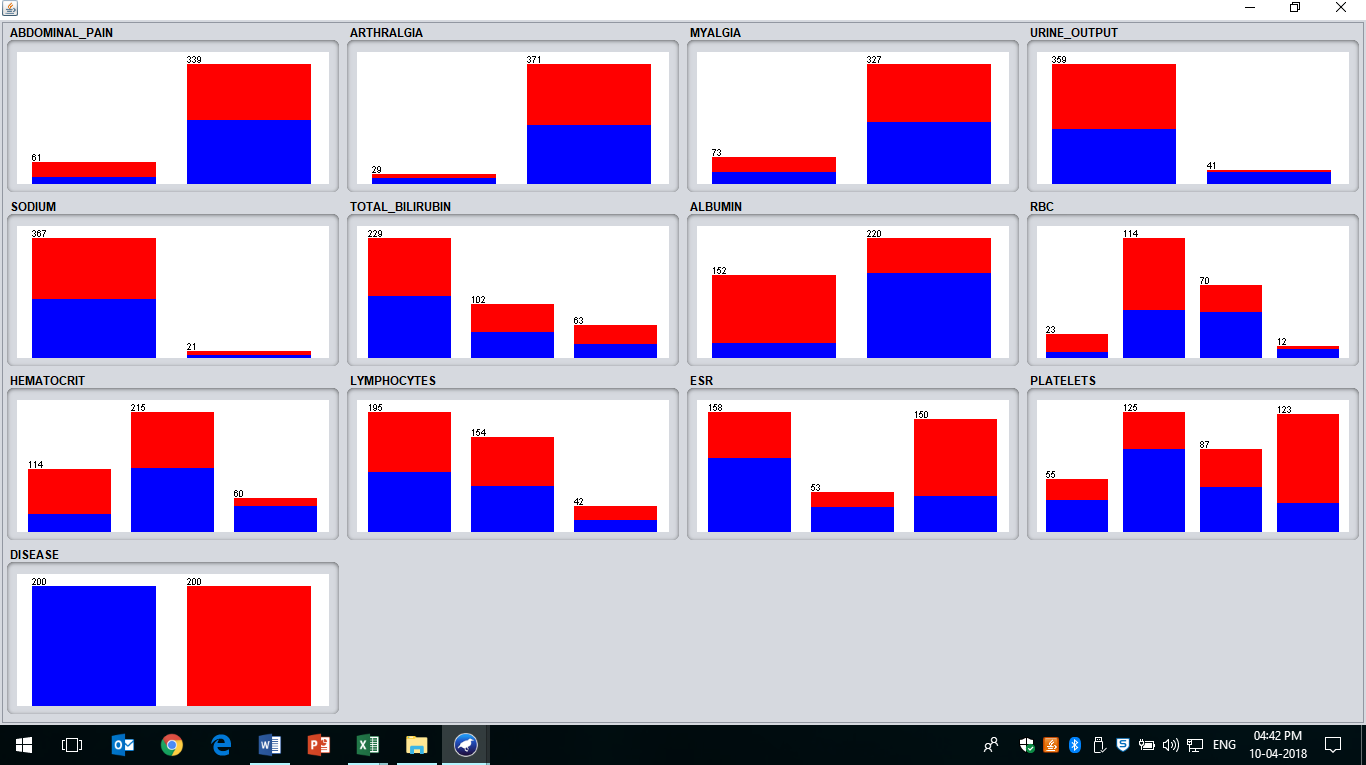


| Correctly Classified Instances | 103 | **75.73 %** |
| --- | --- | --- |
| Incorrectly Classified Instances | 33 | 24.26 % |
| Total Number of Instances | 136 |  |

**10. Malaria vs. Scrub Typhus**


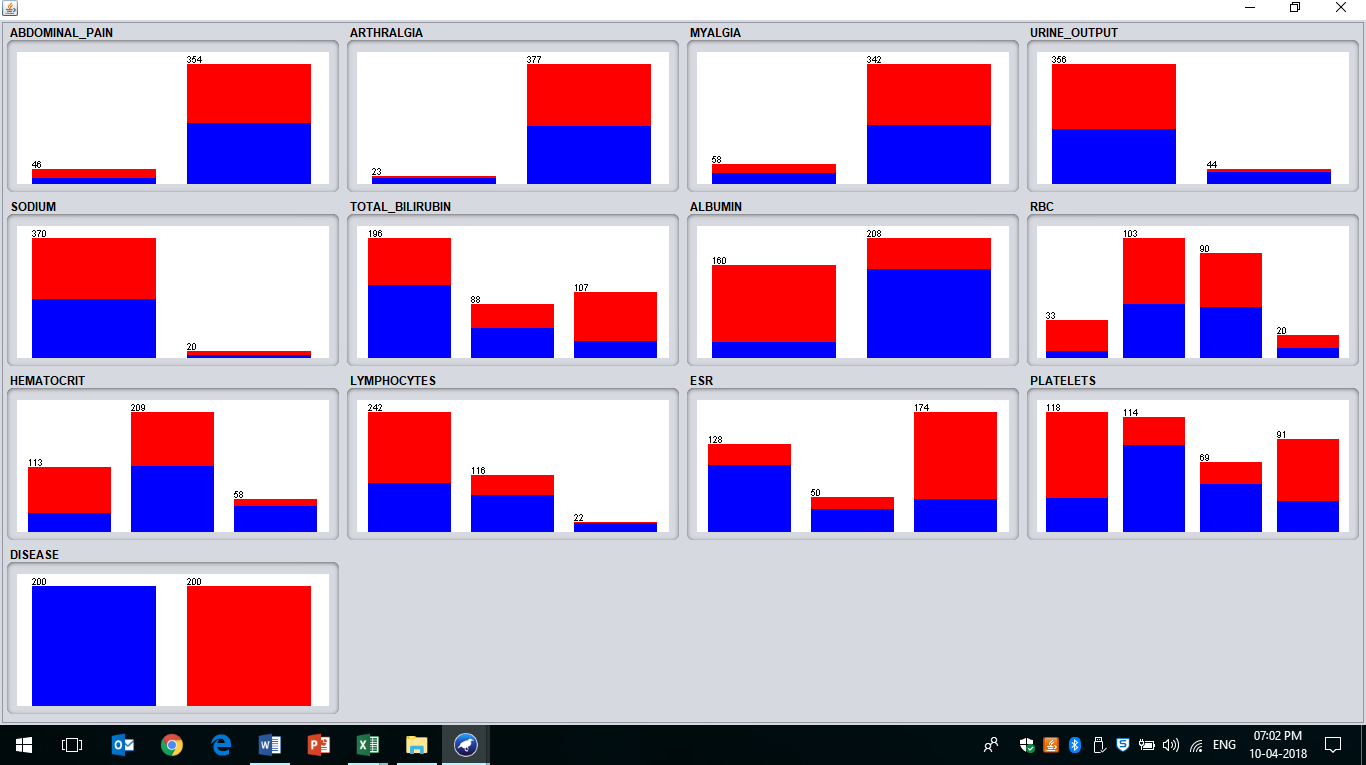


| Correctly Classified Instances | 119 | **87.50 %** |
| --- | --- | --- |
| Incorrectly Classified Instances | 17 | 12.50 % |
| Total Number of Instances | 136 |  |
