# Supplementary material for: Artificial intelligence in differentiating tropical infections: A step ahead
Source: PLoS Negl Trop Dis. 2022 Jun 30;16(6):e0010455. doi: 10.1371/journal.pntd.0010455 (PMC9246149; doi:10.1371/journal.pntd.0010455)
Supplement: S2 File — (DOCX) [file pntd.0010455.s002.docx]

**Supplementary file S2: The data Visualization and parameters with respect to the disease variable**

Data Exploration:


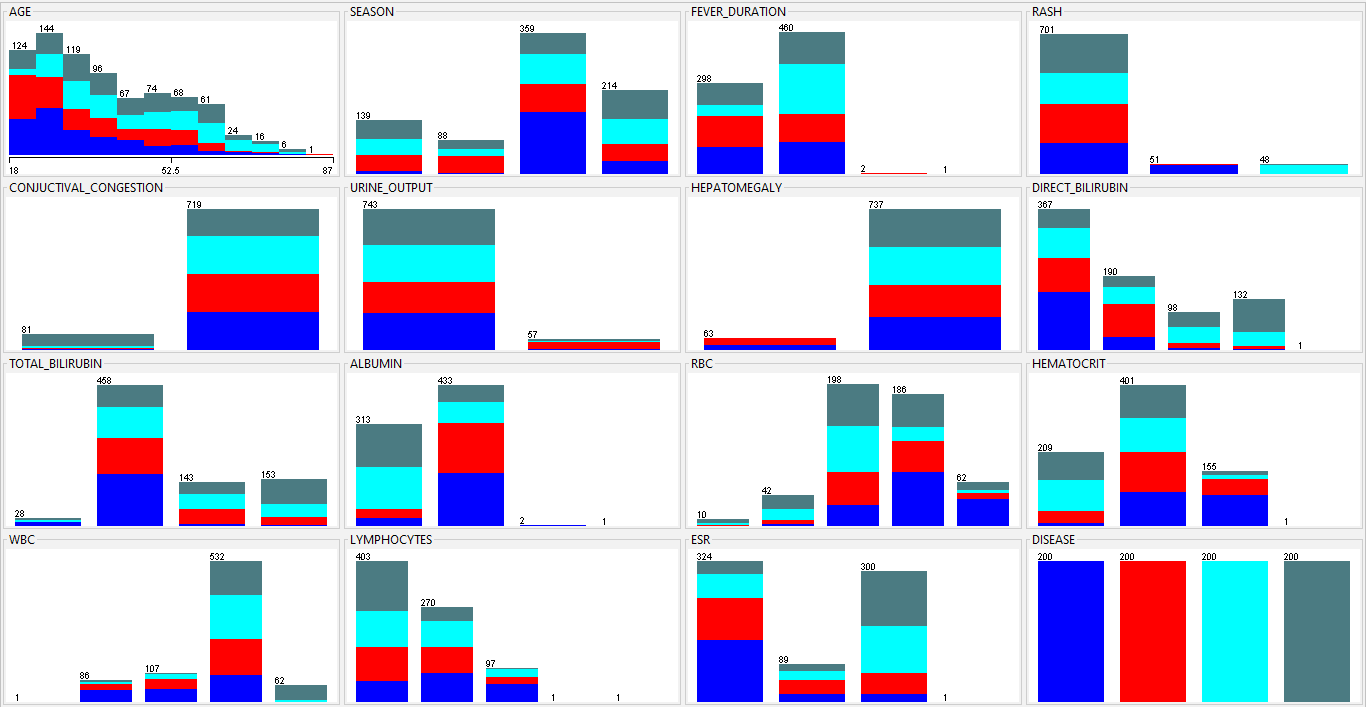


1. Multi class classification

Test Method: Training Set

| **Classifier** | **TP Rate/ sensitivity/ Recall** | **FP Rate** | **Precision/PPV** | **F-Measure** | **ROC Area** |
| --- | --- | --- | --- | --- | --- |
| Naïve Bayes | 0.75 | 0.08 | 0.75 | 0.75 | 0.96 |
| K-NN | 1.0 | 1.0 | 1.0 | 1.0 | 1.0 |
| Multi Layer Perceptron | 0.99 | 0.001 | 0.99 | 0.99 | 0.99 |
| J48 Decision Tree | 0.81 | 0.06 | 0.81 | 0.81 | 0.95 |
| Random Forest | 1.0 | 1.0 | 1.0 | 1.0 | 1.0 |
| Multnomial Logistic Regression | 0.79 | 0.06 | 0.79 | 0.79 | 0.95 |
| Adaboost | 0.39 | 0.20 | NA | NA | 0.68 |

Test Method: 10-fold cross validation

| **Classifier** | **TP Rate/ sensitivity/ Recall** | **FP Rate** | **Precision/PPV** | **F-Measure** | **ROC Area** |
| --- | --- | --- | --- | --- | --- |
| Naïve Bayes | 0.735 | 0.088 | 0.734 | 0.734 | 0.916 |
| K-NN | 0.663 | 0.113 | 0.669 | 0.661 | 0.859 |
| Multi Layer Perceptron | 0.714 | 0.095 | 0.714 | 0.714 | 0.897 |
| J48 Decision Tree | 0.628 | 0.124 | 0.629 | 0.628 | 0.795 |
| Random Forest | 0.688 | 0.104 | 0.687 | 0.687 | 0.891 |
| Multinomial Logistic Regression | 0.70 | 0.09 | 0.70 | 0.70 | 0.90 |
| Adaboost | 0.38 | 0.20 | 0.35 | 0.31 | 0.65 |

1. Dengue Vs Others

Test Method: Training Set

| **Classifier** | **TP Rate/ sensitivity/ Recall** | **FP Rate** | **Precision/PPV** | **F-Measure** | **ROC Area** |
| --- | --- | --- | --- | --- | --- |
| Naïve Bayes | 0.88 | 0.14 | 0.88 | 0.88 | 0.95 |
| K-NN |  |  |  |  |  |
| Multi Layer Perceptron | 0.99 | 0.01 | 0.99 | 0.99 | 0.99 |
| J48 Decision Tree | 0.91 | 0.19 | 0.91 | 0.91 | 0.92 |
| Random Forest | 1.0 | 0 | 1.0 | 1.0 | 1.0 |
| Multinomial Logistic Regression | 0.91 | 0.14 | 0.91 | 0.91 | 0.97 |
| Adaboost | 0.84 | 0.33 | 0.84 | 0.84 | 0.91 |

Test Method: 10 fold cross validation

| **Classifier** | **TP Rate/ sensitivity/ Recall** | **FP Rate** | **Precision/PPV** | **F-Measure** | **ROC Area** |
| --- | --- | --- | --- | --- | --- |
| Naïve Bayes | 0.87 | 0.16 | 0.88 | 0.87 | 0.94 |
| K-NN | 0.88 | 0.22 | 0.87 | 0.88 | 0.91 |
| Multi Layer Perceptron | 0.90 | 0.18 | 0.89 | 0.89 | 094 |
| J48 Decision Tree | 0.84 | 0.29 | 0.84 | 0.84 | 0.86 |
| Random Forest | 0.89 | 0.25 | 0.89 | 0.89 | 0.94 |
| Multinomial Logistic Regression | 0.90 | 0.17 | 0.90 | 0.90 | 0.95 |
| Adaboost | 0.85 | 0.31 | 0.84 | 0.84 | 0.87 |

1. MALARIA Vs Others

Test Method: Training Set

| **Classifier** | **TP Rate/ sensitivity/ Recall** | **FP Rate** | **Precision/PPV** | **F-Measure** | **ROC Area** |
| --- | --- | --- | --- | --- | --- |
| Naïve Bayes | 0.82 | 0.26 | 0.83 | 0.82 | 0.89 |
| K-NN | 0.87 | 0.29 | 0.87 | 0.87 | 0.92 |
| Multi Layer Perceptron | 1.0 | 1.0 | 1.0 | 1.0 | 1.0 |
| J48 Decision Tree | 0.89 | 0.25 | 0.89 | 0.89 | 0.91 |
| Random Forest | 1.0 | 0 | 1.0 | 1.0 | 10 |
| Multinomial Logistic Regression | 0.884 | 0.235 | 0.88 | 0.88 | 092 |
| Adaboost | 0.79 | 0.59 | 0.83 | 0.74 | 0.84 |

Test Method: 10 fold cross validation

| **Classifier** | **TP Rate/ sensitivity/ Recall** | **FP Rate** | **Precision/PPV** | **F-Measure** | **ROC Area** | **Classifier** |
| --- | --- | --- | --- | --- | --- | --- |
| Naïve Bayes | 0.81 | 0.29 | 082 | 0.81 | 0.81 | 0.86 |
| K-NN | 0.82 | 0.4 | 0.81 | 0.82 | 0.81 | 0.84 |
| Multi Layer Perceptron | 0.83 | 0.27 | 0.83 | 0.83 | 0.83 | 0.87 |
| J48 Decision Tree | 0.80 | 0.41 | 0.79 | 0.8 | 0.79 | 0.77 |
| Random Forest | 0.84 | 0.43 | 0.84 | 0.84 | 0.82 | 0.87 |
| Multinomial Logistic Regression | 0.84 | 0.3 | 0.84 | 0.84 | 0.84 | 0.88 |
| Adaboost | 0.79 | 0.53 | 0.78 | 0.79 | 0.76 | 0.82 |

1. SCRUB TYPHUS Vs Others

Test Method: Training Set

| **Classifier** | **TP Rate/ sensitivity/ Recall** | **FP Rate** | **Precision/PPV** | **F-Measure** | **ROC Area** |
| --- | --- | --- | --- | --- | --- |
| Naïve Bayes | 0.8 | 0.27 | 0.81 | 0.81 | 0.87 |
| K-NN | 0.85 | 0.29 | -.85 | 0.85 | 0.9 |
| Multi Layer Perceptron | 0.99 | 0.01 | 0.99 | 0.99 | 0.98 |
| J48 Decision Tree | 0.87 | 0.29 | 0.87 | 0.86 | 0.86 |
| Random Forest | 1.0 | 0 | 1.0 | 1.0 | 1.0 |
| Multinomial Logistic Regression | 0.85 | 0.3 | 0.85 | 0.85 | 0.91 |
| Adaboost | 0.82 | 0.45 | 0.82 | 0.8 | 0.85 |

Test Method: 10 fold cross validation

| **Classifier** | **TP Rate/ sensitivity/ Recall** | **FP Rate** | **Precision/PPV** | **F-Measure** | **ROC Area** |
| --- | --- | --- | --- | --- | --- |
| Naïve Bayes | 0.79 | 0.31 | 0.8 | 0.8 | 0.85 |
| K-NN | 0.8 | 0.38 | 0.79 | 0.79 | 0.8 |
| Multi Layer Perceptron | 0.811 | 0.346 | 0.8 | 0.8 | 0.85 |
| J48 Decision Tree | 0.81 | 0.39 | 0.79 | 0.8 | 0.77 |
| Random Forest | 0.81 | 0.47 | 0.81 | 0.79 | 0.86 |
| Multinomial Logistic Regression | 0.82 | 0.34 | 0.82 | 0.82 | 0.87 |
| Adaboost | 0.82 | 0.44 | 0.81 | 0.8 | 0.81 |

1. LEPTOSYROSYS Vs Others

Test Method: Training Set

| **Classifier** | **TP Rate/ sensitivity/ Recall** | **FP Rate** | **Precision/PPV** | **F-Measure** | **ROC Area** |
| --- | --- | --- | --- | --- | --- |
| Naïve Bayes | 0.85 | 0.2 | 0.86 | 0.85 | 0.91 |
| K-NN | 0.87 | 0.27 | 0.87 | 0.86 | 0.94 |
| Multi Layer Perceptron | 0,99 | 0.91 | 0.99 | 0.99 | 0.99 |
| J48 Decision Tree | 0.91 | 0.19 | 0.91 | 0.91 | 0.92 |
| Random Forest | 1.0 | 0 | 1.0 | 1.0 | 1.0 |
| Multinomial Logistic Regression | 0.89 | 0.2 | 0.897 | 0.89 | 0.94 |
| Adaboost | 0.87 | 0.29 | 0.87 | 0.86 | 0.9 |

Test Method: 10 fold cross validation

| **Classifier** | **TP Rate/ sensitivity/ Recall** | **FP Rate** | **Precision/PPV** | **F-Measure** | **ROC Area** |
| --- | --- | --- | --- | --- | --- |
| Naïve Bayes | 0.84 | 0.21 | 0.85 | 0.84 | 0.9 |
| K-NN | 0.84 | 0.34 | 0.83 | 0.83 | 0.88 |
| Multi Layer Perceptron | 0.85 | 0.27 | 0.84 | 0.84 | 0.89 |
| J48 Decision Tree | 0.85 | 0.31 | 0.84 | 0.84 | 0.82 |
| Random Forest | 0.87 | 0.3 | 0.86 | 0.86 | 0.91 |
| Multinomial Logistic Regression | 0.86 | 025 | 0.86 | 0.86 | 0.9 |
| Adaboost | 0.85 | 0.33 | 0.85 | 0.84 | 0.87 |

F DENGUE Vs MALARIA

Test Method: Training Set

| **Classifier** | **TP Rate/ sensitivity/ Recall** | **FP Rate** | **Precision/PPV** | **F-Measure** | **ROC Area** |
| --- | --- | --- | --- | --- | --- |
| Naïve Bayes | 0.87 | 0.12 | 0.87 | 0.87 | 0.93 |
| K-NN | 0.85 | 0.14 | 0.85 | 0.85 | 0.93 |
| Multi Layer Perceptron | 0.99 | 0.0 | 0.99 | 0.99 | 0.99 |
| J48 Decision Tree | 0.87 | 0.12 | 0.87 | 0.87 | 0.91 |
| Random Forest | 1.0 | 0.0 | 1.0 | 1.0 | 1.0 |
| Multinomial Logistic Regression | 0.89 | 0.1 | 0.89 | 0.89 | 0.96 |
| Adaboost | 0.78 | 0.21 | 0.78 | 0.78 | 0.87 |

Testing Method: 10-fold cross validation

| **Classifier** | **TP Rate/ sensitivity/ Recall** | **FP Rate** | **Precision/PPV** | **F-Measure** | **ROC Area** |
| --- | --- | --- | --- | --- | --- |
| Naïve Bayes | 0.83 | 0.16 | 0.83 | 0.83 | 0.91 |
| K-NN | 0.8 | 0.19 | 0.81 | 0.80 | 0.87 |
| Multi Layer Perceptron | 0.83 | 0.16 | 0.83 | 0.83 | 0.9 |
| J48 Decision Tree | 0.77 | 0.22 | 0.77 | 0.77 | 0.8 |
| Random Forest | 0.82 | 0.17 | 0.82 | 0.82 | 0.9 |
| Multinomial Logistic Regression | 0.85 | 0.15 | 0.85 | 0.85 | 0.91 |
| Adaboost | 0.75 | 0.24 | 0.75 | 0.75 | 0.83 |

G DENGUE Vs SCRUB TYPHUS

Testing Method: Training Set

| **Classifier** | **TP Rate** | **FP Rate** | **Precision** | **F-Measure** | **ROC Area** |
| --- | --- | --- | --- | --- | --- |
| Naïve Bayes | 0.92 | 0.07 | 0.92 | 0.92 | 0.98 |
| K-NN | 0.89 | 0.10 | 0.89 | 0.89 | 0.97 |
| Multi Layer Perceptron | 0.99 | 0.0 | 0.99 | 0.99 | 0.99 |
| J48 Decision Tree | 0.95 | 0.04 | 0.95 | 0.95 | 0.98 |
| Random Forest | 1.0 | 0.0 | 1.0 | 1.0 | 1.0 |
| Multinomial Logistic Regression | 0.97 | 0.03 | 0.97 | 0.97 | 0.99 |
| Adaboost | 0.88 | 0.11 | 0.88 | 0.88 | 0.96 |

Testing Method: 10-fold cross validation

| **Classifier** | **TP Rate/ sensitivity/ Recall** | **FP Rate** | **Precision/PPV** | **F-Measure** | **ROC Area** |
| --- | --- | --- | --- | --- | --- |
| Naïve Bayes | 0.90 | 0.09 | 0.90 | 0.90 | 0.97 |
| K-NN | 0.86 | 0.13 | 0.86 | 0.86 | 0.94 |
| Multi Layer Perceptron | 0.87 | 0.13 | 0.87 | 0.87 | 0.95 |
| J48 Decision Tree | 0.88 | 0.12 | 0.88 | 0.88 | 0.91 |
| Random Forest | 0.90 | 0.09 | 0.90 | 0.90 | 0.96 |
| Multinomial Logistic Regression | 0.90 | 0.10 | 0.90 | 0.90 | 0.94 |
| Adaboost | 0.84 | 0.15 | 0.84 | 0.84 | 0.91 |

H DENGUE Vs LEPTPSYROSIS

Test Method: Training Set

| **Classifier** | **TP Rate/ sensitivity/ Recall** | **FP Rate** | **Precision/PPV** | **F-Measure** | **ROC Area** |
| --- | --- | --- | --- | --- | --- |
| Naïve Bayes | 0.89 | 0.10 | 0.89 | 0.89 | 0.96 |
| K-NN | 0.90 | 0.09 | 0.90 | 0.90 | 0.97 |
| Multi Layer Perceptron | 0.99 | 0.00 | 0.99 | 0.99 | 0.99 |
| J48 Decision Tree | 0.91 | 0.09 | 0.91 | 0.91 | 0.95 |
| Random Forest | 01.0 | 0.0 | 1.0 | 1.0 | 1.0 |
| Multinomial Logistic Regression | 0.94 | 0.05 | 0.94 | 0.94 | 0.98 |
| Adaboost | 0.88 | 0.12 | 0.88 | 0.88 | 0.94 |

Testing Method: 10-fold cross validation

| **Classifier** | **TP Rate/ sensitivity/ Recall** | **FP Rate** | **Precision/PPV** | **F-Measure** | **ROC Area** |
| --- | --- | --- | --- | --- | --- |
| Naïve Bayes | 0.87 | 0.12 | 0.87 | 0.87 | 0.95 |
| K-NN | 0.86 | 0.14 | 0.86 | 0.86 | 0.92 |
| Multi Layer Perceptron |  |  |  |  |  |
| J48 Decision Tree | 0.82 | 0.17 | 0.82 | 0.82 | 0.86 |
| Random Forest | 0.87 | 0.12 | 0.87 | 0.87 | 0.95 |
| Multinomial Logistic Regression | 0.86 | 0.13 | 0.86 | 0.86 | 0.92 |
| Adaboost | 0.84 | 0.15 | 0.84 | 0.84 | 0.92 |

I MALARIA Vs SCRUB TYPHUS

Test Method: Training Set

| **Classifier** | **TP Rate** | **FP Rate** | **Precision** | **F-Measure** | **ROC Area** |
| --- | --- | --- | --- | --- | --- |
| Naïve Bayes | 0.87 | 0.12 | 0.87 | 0.87 | 0.94 |
| K-NN | 0.89 | 0.10 | 0.89 | 0.89 | 0.94 |
| Multi Layer Perceptron | 1.0 | 0.0 | 1.0 | 1.0 | 1.0 |
| J48 Decision Tree | 0.88 | 0.12 | 0.88 | 0.88 | 0.93 |
| Random Forest | 1.0 | 0.0 | 1.0 | 1.0 | 1.0 |
| Multinomial Logistic Regression | 0.90 | 0.09 | 0.90 | 0.90 | 0.97 |
| Adaboost | 0.83 | 0.16 | 0.83 | 0.83 | 0.93 |

Training Method: 10-fold cross validation

| **Classifier** | **TP Rate/ sensitivity/ Recall** | **FP Rate** | **Precision/PPV** | **F-Measure** | **ROC Area** |
| --- | --- | --- | --- | --- | --- |
| Naïve Bayes | 0.85 | 0.14 | 0.85 | 0.85 | 0.93 |
| K-NN | 083 | 0.17 | 0.83 | 0.83 | 0.90 |
| Multi Layer Perceptron | 0.83 | 0.16 | 0.83 | 0.83 | 0.92 |
| J48 Decision Tree | 0.81 | 0.18 | 0.81 | 0.81 | 0.84 |
| Random Forest | 0.84 | 0.15 | 0.84 | 0.84 | 0.92 |
| Multinomial Logistic Regression | 0.84 | 0.15 | 0.84 | 0.84 | 0.91 |

J MALARIA Vs LEPTOSYROSIS

Testing Method: Training Set

| **Classifier** | **TP Rate/ sensitivity/ Recall** | **FP Rate** | **Precision/PPV** | **F-Measure** | **ROC Area** |
| --- | --- | --- | --- | --- | --- |
| Naïve Bayes | 0.89 | 0.10 | 0.89 | 0.89 | 0.96 |
| K-NN | 0.90 | 0.09 | 0.90 | 0.90 | 0.97 |
| Multi Layer Perceptron | 0.99 | 0.00 | 0.99 | 0.99 | 0.99 |
| J48 Decision Tree | 0.91 | 0.09 | 0.91 | 0.91 | 0.95 |
| Random Forest | 1.0 | 0.0 | 1.0 | 1.0 | 1.0 |
| Multinomial Logistic Regression | 0.94 | 0.05 | 0.94 | 0.94 | 0.98 |
| Adaboost | 0.88 | 0.11 | 0.88 | 0.88 | 0.94 |

Testing Method: 10-fold cross validation

| **Classifier** | **TP Rate/ sensitivity/ Recall** | **FP Rate** | **Precision/PPV** | **F-Measure** | **ROC Area** |
| --- | --- | --- | --- | --- | --- |
| Naïve Bayes | 0.87 | 0.12 | 0.87 | 0.87 | 0.95 |
| K-NN | 0.86 | 0.14 | 0.86 | 0.86 | 0.92 |
| Multi Layer Perceptron | 0.87 | 0.12 | 0.87 | 0.87 | 0.94 |
| J48 Decision Tree | 0.82 | 0.17 | 0.82 | 0.82 | 0.86 |
| Random Forest | 0.87 | 0.12 | 0.87 | 0.87 | 0.95 |
| Multinomial Logistic Regression | 0.86 | 0.13 | 0.86 | 0.86 | 0.92 |
| Adaboost | 0.84 | 0.15 | 0.84 | 0.84 | 0.92 |

K SCRUB TYPHUS Vs LEPTOSYROSIS

Testing Method: Training Set

| **Classifier** | **TP Rate/ sensitivity/ Recall** | **FP Rate** | **Precision/PPV** | **F-Measure** | **ROC Area** |
| --- | --- | --- | --- | --- | --- |
| Naïve Bayes | 0.80 | 0.19 | 0.80 | 0.80 | 0.89 |
| K-NN | 0.79 | 0.20 | 0.79 | 0.79 | 0.89 |
| Multi Layer Perceptron | 1.0 | 0.0 | 1.0 | 1.0 | 1.0 |
| J48 Decision Tree | 0.85 | 0.14 | 0.85 | 0.85 | 0.92 |
| Random Forest | 1.0 | 0.0 | 1.0 | 1.0 | 1.0 |
| Multinomial Logistic Regression | 0.84 | 0.15 | 0.84 | 084 | 0.93 |
| Adaboost | 0.79 | -.20 | 0.79 | 0.79 | 0.87 |

Testing Method: 10-fold cross validation

| **Classifier** | **TP Rate/ sensitivity/ Recall** | **FP Rate** | **Precision/PPV** | **F-Measure** | **ROC Area** |
| --- | --- | --- | --- | --- | --- |
| Naïve Bayes | 0.77 | 0.22 | 0.77 | 0.77 | 0.86 |
| K-NN | 0.73 | 0.27 | 0.73 | 0.73 | 0.80 |
| Multi Layer Perceptron | 0.77 | 0.23 | 0.77 | 0.77 | 0.84 |
| J48 Decision Tree | 0.73 | 0.26 | 0.73 | 0.73 | 0.78 |
| Random Forest | 0.78 | 0.22 | 0.78 | 0.78 | 0.85 |
| Multinomial Logistic Regression | 0.77 | 0.22 | 0.77 | 0.77 | 0.85 |
| Adaboost | 0.77 | 0.22 | 0.77 | 0.77 | 0.84 |
