# Supplementary material for: Artificial intelligence in differentiating tropical infections: A step ahead
Source: PLoS Negl Trop Dis. 2022 Jun 30;16(6):e0010455. doi: 10.1371/journal.pntd.0010455 (PMC9246149; doi:10.1371/journal.pntd.0010455)
Supplement: S3 File — (DOCX) [file pntd.0010455.s003.docx]

**Supplementary file S3: The effect of age on model performance**

**Age below 26.5**

| Classifier | Accuracy | TP Rate | FP Rate | Precision | Recall | F-Measure | ROC Area |
| --- | --- | --- | --- | --- | --- | --- | --- |
| Random Forest | 67.62 | 0.67 | 0.10 | 0.67 | 0.67 | 0.67 | 0.89 |
| Multinomial Logistic Regression | 70.87 | 0.70 | 0.09 | 0.70 | 0.70 | 0.70 | 0.90 |
| Multi Layer Perceptron | 69.12 | 0.69 | 0.10 | 0.69 | 0.69 | 0.69 | 0.89 |

**Age between 26.5 and 36.5**

| Classifier | Accuracy | TP Rate | FP Rate | Precision | Recall | F-Measure | ROC Area |
| --- | --- | --- | --- | --- | --- | --- | --- |
| Random Forest | 69 | 0.69 | 0.10 | 0.69 | 0.69 | 0.69 | 0.89 |
| Multinomial Logistic Regression | 69.75 | 0.69 | 0.10 | 0.69 | 0.69 | 0.69 | 0.90 |
| Multi Layer Perceptron | 69.62 | 0.69 | 0.10 | 0.69 | 0.69 | 0.69 | 0.89 |

**Age between 36.5 and 50.5**

| Classifier | Accuracy | TP Rate | FP Rate | Precision | Recall | F-Measure | ROC Area |
| --- | --- | --- | --- | --- | --- | --- | --- |
| Random Forest | 69.25 | 0.69 | 0.10 | 0.69 | 0.69 | 0.69 | 0.88 |
| Multinomial Logistic Regression | 69 | 0.69 | 0.10 | 0.68 | 0.69 | 0.69 | 0.90 |
| Multi Layer Perceptron | 68.87 | 0.68 | 0.10 | 0.68 | 0.68 | 0.68 | 0.88 |

**Age between 50.5 and 80**

| Classifier | Accuracy | TP Rate | FP Rate | Precision | Recall | F-Measure | ROC Area |
| --- | --- | --- | --- | --- | --- | --- | --- |
| Random Forest | 68.87 | 0.68 | 0.10 | 0.68 | 0.68 | 0.68 | 0.89 |
| Multinomial Logistic Regression | 70.75 | 0.70 | 0.09 | 0.70 | 0.70 | 0.70 | 0.90 |
| Multi Layer Perceptron | 69.25 | 0.69 | 0.10 | 0.69 | 0.69 | 0.69 | 0.89 |
